# UCSF’S EMANCIPATORY SCIENCES LAB AS PRAXIS: TOWARD THEORY AND ACTION

**DOI:** 10.1093/geroni/igae098.1742

**Published:** 2024-12-31

**Authors:** Brittney Pond

**Affiliations:** University of California San Francisco, San Francisco, California, United States

## Abstract

This presentation discusses the UCSF Emancipatory Sciences Lab as a model for intergenerational, international, and cross-institutional collaboration to advance public scholarship centering anti-oppression and healing oriented research, policy, and practice. The lab promotes participation in public scholarship and activism and currently has over 140 members. The Emancipatory Sciences Lab highlights the importance of public scholarship via the production of a podcast, blog series and posts, and engagement on social media including X (formerly Twitter) and LinkedIn. Our podcast, titled EDGES: Engaging Dialogues Generating Emancipatory Sciences, interviews scholars, practitioners, artists, students, and activists to elevate their work and share critical discussions with a broader audience. The Emancipatory Sciences Lab provides tools, resources, and spaces for people within and beyond academia to engage in collaborative work with scholars, practitioners, and students. One example is the Student Writing Series that I created and facilitated, which has provided a monthly space for students to set writing goals, co-write together, and share resources for writing for nearly one year. The Emancipatory Sciences Lab website features a searchable clearinghouse of publications for people in our network in the emancipatory sciences, which is valuable in providing resources and elevating critical work. We host webinar series and promote events from folks in our network to provide space for collaboration, discussion, and learning. Given my background as a first-generation scholar from a low socioeconomic status community, I am excited to share about the work stemming from the Emancipatory Sciences Lab that creates collaborative spaces to share and learn.

